# Effects of Limiting the Number of Different Cross-Sections Used in Statically Loaded Truss Sizing and Shape Optimization

**DOI:** 10.3390/ma17061390

**Published:** 2024-03-18

**Authors:** Nenad Kostić, Nenad Petrović, Vesna Marjanović, Ružica R. Nikolić, Janusz Szmidla, Nenad Marjanović, Robert Ulewicz

**Affiliations:** 1 University of Kragujevac, Faculty of Engineering, 34000 Kragujevac, Serbia; nkostic@kg.ac.rs (N.K.); npetrovic@kg.ac.rs (N.P.); vmarjanovic@kg.ac.rs (V.M.); nesam@kg.ac.rs (N.M.); 2 Research Centre, University of Zilina, 01026 Zilina, Slovakia; 3 Department of Mechanics and Machine Design Fundamentals, Częstochowa University of Technology, 42-201 Częstochowa, Poland; janusz.szmidla@pcz.pl; 4 Department of Production Engineering and Safety, Czestochowa University of Technology, 42-201 Czestochowa, Poland

**Keywords:** truss optimization, cardinality, Euler buckling, optimization constraints, sizing and shape optimization

## Abstract

This research aims to show the effects of adding cardinality constraints to limit the number of different cross-sections used in simultaneous sizing and shape optimization of truss structures. The optimal solutions for sizing and shape optimized trusses result in a generally high, and impractical, number of different cross-sections being used. This paper presents the influence of constraining the number of different cross-sections used on the optimal results to bring the scientific results closer to the applicable results. The savings achieved using the cardinality constraint are expected to manifest in more than just the minimization of weight but in all the other aspects of truss construction, such as labor, assembly time, total weld length, surface area to be treated, transport, logistics, and so on. It is expected that the optimal weight of the structures would be greater than when not using this constraint; however, it would still be below conventionally sized structures and have the added benefits derived from the simplicity and elegance of the solution. The results of standard test examples for each different cardinality constraint value are shown and compared to the same examples using only a single cross-section on all bars and the overall optimal solution, which does not have the cardinality constraint. An additional comparison is made with results of just the sizing optimization from previously published research where authors first used the same cardinality constraint.

## 1. Introduction

Advancements in structural truss optimization, since the start of the digital age, were largely limited in complexity by the processing power and storage capacity of the computers available in the past. To have the optimal solutions made applicable in the real world, the gradual addition of constraints, which kept up with increases in computational speed and storage space, has allowed for results that are closer and closer to being directly used. Furthermore, this can now be carried out without the need for additional revisions and modifications, which would require skilled and experienced engineers.

The first optimization results were obtained only for the simple structures; they used continuous variables for sizing, resulting in designs that were practically impossible to construct. The sizes are generally allowed to vary within a millimeter or smaller range, which would be prohibitively expensive to produce and require very narrow tolerances since these structures are initially optimized without a safety factor, and any dimensional variation could result in structural failure. In the vast majority of cases, truss members are assembled from standard cross-section elements, which are produced in a specific set of different sizes. Due to production limitations, these bars are also made within specific dimensional tolerances. Adding discrete variables for cross-sections was the first step in bringing the optimal solutions closer to applicable results.

Researchers in [1] used continuous sizing variables, as recently as 2015, to show the efficacy of their algorithm on truss examples. The change to discrete cross-section variables results in greater optimal weights, as presented in [2,3]. The authors of [4] developed an optimization algorithm for more efficient truss optimization, tested on a continuous and a discrete variable cross-section problem of a 25-bar truss.

In the past few decades, the use of dynamic buckling constraints has become increasingly prevalent as these are the constraints that drastically increase the complexity of the problem. These are nonlinear constraints and result in creating a non-convex feasible solution domain. The use of evolutionary-based algorithms has allowed these problems to be solved and able to avoid being trapped in local optima. Even with improved algorithms, many papers, such as [5], consider fixed values for buckling constraints, even though this does not guarantee applicable results, or even the minimal possible weight while maintaining stability. Dynamic buckling constraints were used by authors in [6] to demonstrate the improvement in achieving the minimal weight using their proposed algorithm and compared their results to [7], which used these constraints on various test examples. A sizing optimization comparison, for example, with the Euler buckling constraint turned on and off in [8], shows the drastic increase in optimal weight due to the increase in the cross-sections of the compressed bars. The authors presented a new algorithm called PO (political optimization) in [9] and successfully used it to find the optimal solutions in standard test examples with the addition of buckling constraints. This paper uses continuous cross-section sizing variables, making the results inapplicable in practice. The authors in [10,11,12,13] also successfully showed the implementation of Euler buckling in truss optimization problems.

Recently, the authors in [14,15,16,17,18,19] used different approaches to add a cardinality constraint to truss optimization models. Researchers in [14] considered static loading problems by applying buckling constraints to compressed bars for sizing optimization. The researchers in [15] considered the effects of dynamic actions introducing natural frequency constraints in sizing and shape optimization, which were influenced by work carried out in [18,19]. The authors in [19] considered just sizing as well as simultaneous sizing and shape optimization using typical stress and displacement constraints and added the Euler buckling constraints using a fixed coefficient rather than the moment of inertia, thus showing the effects of limiting the number of different cross-sections being used.

This research aims to bridge the gap between optimization results and applicable structures. Expanding on the previous work, this research also uses minimal element length and dynamic Euler buckling in addition to the standard constraints, as well as recording results for each different number of cross-sections used, at least up to the optimal number when this constraint is not used. Some of the most frequently used standard test examples are used to demonstrate the effects of having cardinality constraints.

The paper is structured as follows: in Section 2, the mathematical formulations of existing, known constraints are formulated in the context of sizing and shape optimization use. Section 3 gives the mathematical and logical explanation of the cardinality constraint. Section 4 shows the configurations for the test examples which were used. The results are presented in Section 5 with comparisons to sizing optimized results from the literature, which also use the cardinality constraint, as well as the overall optimum for each example. Finally, conclusions are drawn in Section 6, giving an overview of what was carried out and suggesting future research directions.

## 2. Simultaneous Truss Sizing and Shape Optimization

Sizing optimization considers cross-sections as variables. This research looks at cross-section variables as a discrete set of values to achieve applicable results. The objective function is to find the combination of cross-sections which give a minimal weight while subjected to stress and displacement constraints. Most truss sizing problems found in the literature view the minimal weight design problem as follows:

(1) $$\left\{\begin{array}{l} minW\left(A\right)=\sum_{i=1}^{i=n}\;\;\rho_{i}\;A_{i}l_{i}\;\mathrm{with}\;A=\left(A_{1},\ldots ,A_{n}\right)\\ \mathrm{subjected}\;\mathrm{to}\;\left\{\begin{array}{l} A_{min}\leq A_{i}\leq A_{max}\;\;\;\mathrm{for}\;i=1,\ldots ,n\\ \sigma_{min}\leq \sigma_{i}\leq \sigma_{max}\;\mathrm{for}\;i=1,\ldots ,n\\ u_{min}\leq u_{j}\leq u_{max}\;\;\;\mathrm{for}\;j=1,\ldots ,k \end{array}\right. \end{array}\right.,$$

where *W* is the weight of the truss, *n* is the number of truss elements, *A_i_* is the area of the *i*^th^ element cross-section, *l_i_* is the length of the *i*^th^ element, *σ_i_* is the stress of the *i*^th^ element, *u_j_* is the displacement of the *j*^th^ node, and *k* is the number of nodes.

This type of optimization has shown the greatest possibility of decreasing the overall weight when compared to shape and topology optimizations individually. In most cases in the literature, the standard test examples used full, round cross-sections. However, any number of different shapes and dimensions of cross-section profiles can be used. This is only transferable to other cross-section shapes for elements in tension. Compressed elements must also be tested for buckling (2). Euler buckling constraints also consider the minimum area moment of inertia, which is why the results of these examples are only useful for the specific profile shape with which they are optimized.

The Euler buckling is added to obtain results that can be applied in practice. The iterative change in the moment of inertia, due to the change in cross-sections, also changes the Euler critical buckling constraint in each iteration (2). This constraint is, therefore, considered to be a dynamic constraint. Its addition significantly increases the complexity of the optimization problem. The constraint used in this research is the Euler critical load, as the stress comparison uses the same area on both sides of the expression.

(2) $$\begin{array}{l} \sigma_{Ai}^{comp}\leq \sigma_{Ki}\\ \mathrm{where}\;\;\;\sigma_{Ai}^{comp}=\frac{F_{Ai}^{comp}}{A_{i}}\;\;\;\mathrm{and}\;\;\;\sigma_{Ki}=\frac{F_{Ki}}{A_{i}}\\ F_{Ki}=\frac{\pi^{2}\;\cdot E_{i}\cdot \;I_{i}}{l_{i}^{2}}\\ \left|F_{Ai}^{comp}\right|\leq F_{Ki}\;\;\mathrm{for}\;\;i=1,\ldots ,n\; \end{array},$$

where *σ_Ai_* is the axial compression stress of the *i*^th^ bar element, and *σ_Ki_* is the critical buckling stress of the *i*^th^ element; $F_{Ai}^{comp}$ is the axial compression force, *F_Ki_* is the Euler’s critical load of the *i*^th^ element, *E_i_* is the *i*^th^ element’s modulus of elasticity *I_i_* is the minimum moment of inertia of the *i*^th^ element’s cross-section, and *l_i_* is the length of the *i*^th^ element. The constraint from Equation (2) is added to the existing constraints from Equation (1).

Shape optimization considers the positions of nodes as variables and allows for drastic changes in shape, which are not always an obvious solution. It is used individually to achieve a lower weight than the initial concept and with a set of previously calculated bar cross-sections. This is a significant limitation, as the changes in shape can decrease stress and, therefore, will result in oversized cross-sections being used, thereby wasting material.

This research also uses a minimal element length constraint to avoid excessively short elements, which would be impractical to implement. The value of each example is determined by experience or design guidelines given in standards or literature. The constraint is formulated as follows:

(3) $$\begin{array}{l} l_{i}\geq l_{\min}\;\;=\;\;\;\;\mathrm{for}\;i\;=1,\;\;\ldots ,\;\;n\\ l_{i}=\sqrt{{\left(x_{ib}-x_{ia}\right)}^{2}+{\left(y_{ib}-y_{ia}\right)}^{2}} \end{array},$$

where element length *l_i_* is from 1 to *n*, which is between nodes *a* and *b* with coordinates ($x_{ia},y_{ia}$) and ($x_{ib},y_{ib}$), respectively. The shape optimization node coordinate constraints implicitly define the maximal element values; therefore, they are unnecessary. If there were a need for a maximal element length constraint, the same method could be used to create it. This could be an interesting constraint for limiting the bar lengths not to exceed the stock lengths to avoid extensions. This constraint supplements the node coordinate bounds to prevent a pileup of nodes close to one location, resulting in excessively short elements. The minimal length of elements also ensures a reasonable amount of space for beam connections and access to the joint for establishing those connections. For example, in the 10-bar truss example in Section 4.1, nodes 1 and 3 could even be located in the same position, resulting in two elements occupying the same space while having one element with a length of 0.

A sequential approach, compared to the simultaneous one explored in [20], showed the benefits of the simultaneous optimization of structural aspects. Using sizing and shape optimization simultaneously is expected to give better results than each individually or even sequentially.

In this research, the same penalty function is assigned to all the constraints. The penalty function multiplies the invalid result by a large number in case any single or multiple constraints were not met.

## 3. Constraining the Number of Different Cross-Sections Used

Previous research [14] has shown that the use of sizing optimization can result in solutions that are impractical to construct. Namely, if sizing optimization results in a truss with a large number of different cross-sections, that solution, though good on paper, would not find application in practice since the logistics and assembly of such a structure would be unnecessarily more complex. This is, therefore, another constraint, which needs to be addressed to achieve actual optimal results, [18]. Adding another constraint drastically increases the complexity of solving the already multi-modal discontinuous function; however, it is paramount for achieving applicable results.

The mathematical formulation for constraining the number of different cross-sections proposed in this paper is given by:

(4) $$\begin{array}{c} |\{A_{1}^{G},A_{2}^{G},A_{3}^{G},\ldots ,A_{n}^{G}\}|\;=m\\ m\;\;\leq \;\;m_{\max}\;\;\;\; \end{array},$$

where $A_{n}^{G}$ is the cross-section geometry area assigned to the *n*^th^ element, *m* is the cardinal of the used cross-section area geometries set, and *m_max_* is the maximal allowed number of different cross-sections.

**Figure 1:**
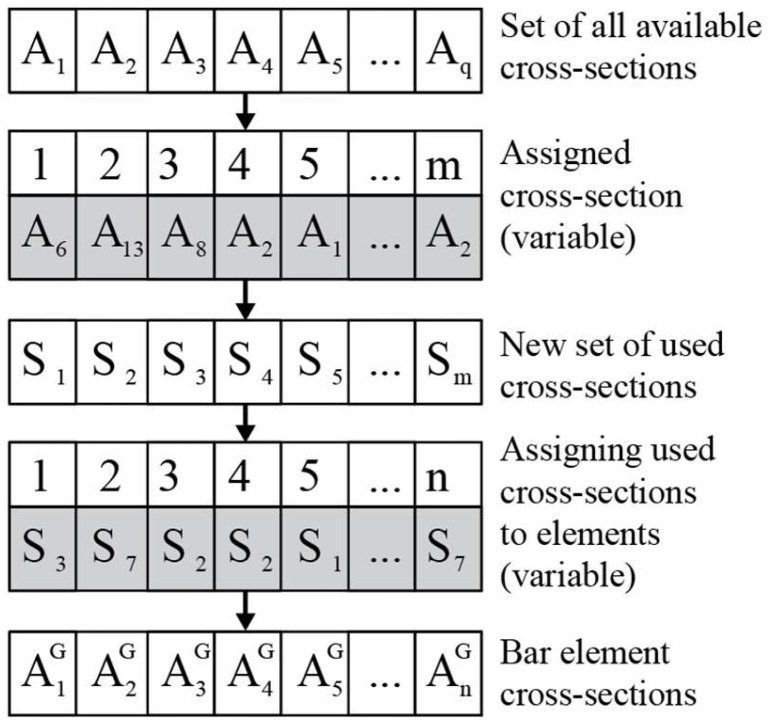
General example of constructing the variable sets.

To implement this constraint, authors have developed an original software in Rhinoceros 6’s Grasshopper and Karamba3D 2.2.0 using two variable sets. One set includes *m* variables, each of which can adopt a cross-section diameter from the discrete set of all available cross-sections. The other set of variables assigns a cross-section to each bar from the previous set of variables, guaranteeing that the constraint set by (4) is always satisfied. This is illustrated in Figure 1 with a generalized example.

In this research, the numbers of different cross-sections used were set as fixed values to see the influence of limiting the number of different cross-sections. The first set of variables was set to have all different cross-sections selected, and the second was forced to have at least one of each different cross-section from the set, thereby changing the inequality in expression (4) to an equality, with *m_max_* different first variables. This was carried out to plot the changes for each number.

## 4. Test Examples

Sizing and shape optimization problems are the most frequently demonstrated in the literature using examples of 10-, 17-, and 25-bar truss problems. Those examples were previously analyzed in [2] and in [14], in particular, where the number of different cross-sections was first constrained for sizing optimization. All examples use dynamic Euler buckling constraints for compressed elements. Adding this constraint ensures that the optimal truss configurations can stay in the elastic zone and maintain stability under the defined loads. This research uses discrete values for the cross-section areas from previously published research. The new cardinality constraint for each example is set to an exact value of different cross-sections allowed for that optimization run. This constraint is suggested to be used to set a maximal, not exact number of cross-sections to be used, as is the case here. The reason for using exact numbers in this research is to show optimal values for each different number to find trends in the change in cardinality. The examples were optimized for each number of different cross-section profiles, which was smaller than the number of cross-sections in the optimal solution and for a few numbers greater than that number. The examples with a greater number of different cross-sections than the optimal ones were included to observe the trend and are not considered useful in practice.

### 4.1. Planar 10-Bar Truss Problem

**Figure 2:**
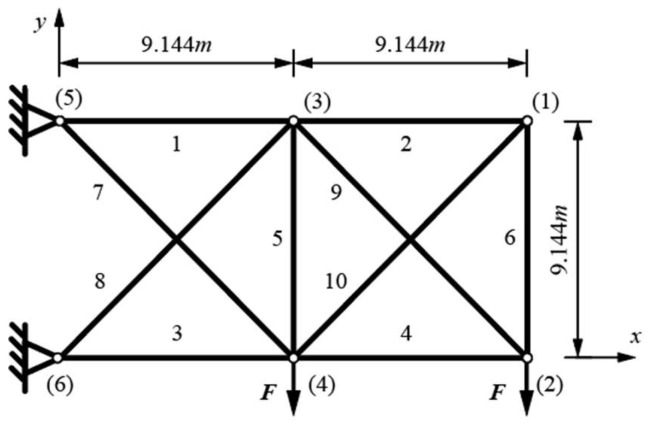
Planar 10-bar truss layout with labeled bars (1–10) and nodes ((1) to (6)).

The planar 10-bar truss is laid out as shown in Figure 2. Bars are made of aluminum 6063-T5, with a Young modulus of 0.7·10^5^ MPa and a density of 2.7·g/cm^3^. A point load is applied to nodes (2) and (4) with a value of *F* = 444.82 kN in the −y direction.

Constraints include a maximal displacement of ±0.0508 m of all nodes in all directions, maximal axial stress of ±172.37 MPa for all bars, and the Euler buckling constraints for all the compressed bars. A discrete set of variables for the full circular cross-sections was used from previously published research. We decided to use the full-circular cross-sections, which are the standard test examples widely recognized in the field, to prove that our research was valid, and with comparable results. The hollow circular cross-sections might be more “real-world’ examples, which we shall consider in future research. There are 50 possible cross-section diameters: 3, 4, 6, 6.5, 7, 7.5, 8, 8.5, 9, 10, 11, 12, 12.5, 14, 15, 16, 17.5, 18, 19, 20, 22.5, 25, 27.5, 28, 30, 31.5, 32.5, 35, 37.5, 40, 42.5, 45, 47.5, 50, 52.5, 55, 57.5, 60, 62.5, 65, 70, 75, 80, 85, 90, 95, 100, 110, 125, given in mm. The coordinates of nodes (3) and (4) are variable in the x and y directions within the bounds of the initial geometry (±9.144 m and −9.144 m in the x direction for nodes (3) and (4), respectively, and −9.144 m in the y direction for both nodes).

### 4.2. Planar 17-Bar Truss Problem

**Figure 3:**
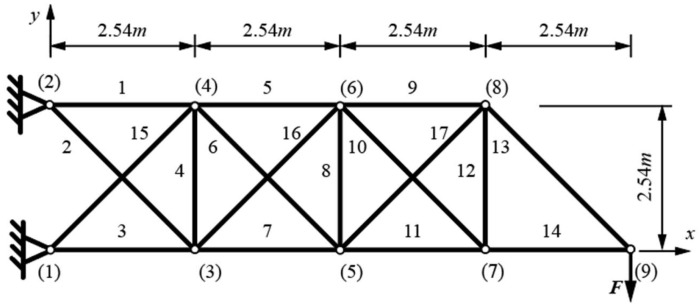
Planar 17-bar truss layout with labeled bars (1–17) and nodes ((1) to (9)).

The planar 17-bar truss problem is laid out as shown in Figure 3. Bars are made of steel with a Young modulus of 2.1 × 10^5^ MPa and a density of 7.4·g/cm^3^. A load of *F* = 444.82 kN is applied in node (9) in the −y direction.

Displacement is constrained to ±0.0508 m for all nodes in both directions, and Euler buckling constraints are used for all compressed bars. The discrete set of variables, for the full circular cross-sections, is used from the previous example. There are 49 possible cross-section diameters, ranging from 6 mm (28.3 mm^2^) to 250 mm (49,089.4 mm^2^) [14]. The coordinates of nodes (3) to (8) are variable in the x and y direction, and the y coordinate of node (9) is variable. Nodes (3) to (8) can all vary from 0 to 10.16 m in the x direction and −2.54 to 5.08 m in the y direction. Node (9) is limited from 0 to 2.54 m in the y direction.

### 4.3. Spatial 25-Bar Truss Problem

**Figure 4:**
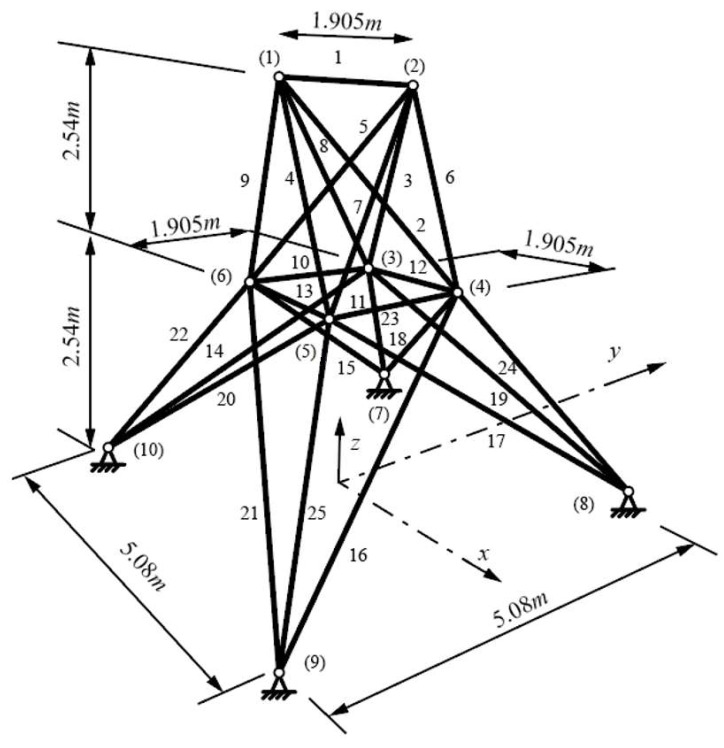
Spatial 25-bar truss layout with labeled bars (1–25) and nodes ((1) to (10)).

The spatial 25-bar truss problem is shown in Figure 4. The material characteristics and the discrete set of variables for full round cross-sections are the same as in the 10-bar truss problem. The force vectors in the nodes are given in (x, y, z) components as follows: node (1) (4.448, −44.48, −44.48) kN, node (2) (0, −44.48, −44.48) kN, node (3) (2.224, 0, 0) kN, and node (6) (2.669, 0, 0) kN. The truss cross-sections are grouped into the following eight sets: 1 (A1), 2 (A2–A5), 3 (A6–A9), 4 (A10–A11), 5 (A12–A13), 6 (A14–A17), 7 (A18–A21), and 8 (A22–A25) [18].

Constraints include a tensile stress limit of 40 kN for all bar groups, a maximal displacement of ± 0.009 m for all nodes in all directions, and Euler buckling constraints used for all compressed bars. The shape variables for this example are as follows: 0.508 m ≤ x_4_, x_5_, −x_3_, −x_6_ ≤ 1.524 m; 1.016 m ≤ y_3_, y_4_, −y_5_, −y_6_ ≤ 2.032 m; 2.286 m ≤ z_3_, z_4_, z_5_, z_6_ ≤ 3.302 m; 1.016 m ≤ x_8_, x_9_, −x_7_, −x_10_ ≤ 2.032 m; and 2.540 m ≤ y_7_, y_8_, −y_9_, −y_10_ ≤ 3.556 m.

## 5. Results

All test examples were optimized in the original software created by the authors, using a genetic algorithm due to its availability and favorable characteristics. Any other heuristic optimization algorithm could be used with this set of constraints. Each example was optimized ten times for each of the specific numbers of different cross-section constraints. The results presented in this research are the best from each set of results. For each example, multiple solutions, of the ten repeated optimizations, for the particular example were close to the best results, showing that the best solutions are near the global optima. It should be noted that the termination criteria (maximal stagnant population of 50), as well as population size (50), maintained a population of 5%, and inbreeding (75%) for each optimization were the same for all examples. Combinatorial methods (derivative-free algorithms) are naturally suited for discrete or integer optimization problems. However, their use can be hampered when the number of design variables increases. In this regard, gradient-based methods are faster but can easily become trapped in local minima. Hence, there is fertile ground for the development of new algorithms. This research focused on the effects of simultaneously size and shape design variables in a discrete–continuous mixed optimization setting using a well-known algorithm.

For each of the models, the solution was represented by one cross-section (the same cross-section for the entire structure), the sizing variables were set to only allow for one cross-section for all bars and simultaneous shape optimization was conducted. Table 1 gives the cross-section areas of the optimal models for 10-bar truss sizing and shape optimization according to the number of different cross-sections used. Note that in the first column the solution with one cross-section is given, where all the elements have the same cross-section is an optimal solution, where sizing only allows for one cross-section in the simultaneous optimization. The parameter *m* is set to be equal to *m_max_*, which in that case is 1. Basically, the whole structure is sized according to the most stressed element in that configuration. The cross-sections are presented by areas, given in cm^2^, and not by diameters. A reason for this is for the sake of comparison, since this is the usual way in which the cross-sections were presented in the vast majority of previous research. That is probably a remnant from the period before the buckling was considered, as in that case any profile with the same area would “work” in these situations, if only it were not for buckling.

Table 2 shows the optimal weights and differences from the solution with a single cross-section and from the optimal solution for the 10-bar truss sizing and shape problem. In this example, the optimal solution uses eight different cross-sections. The optimal coordinates of points 1 and 3 are given in Table 3 for each solution with a different number of cross-sections.

**Figure 5:**
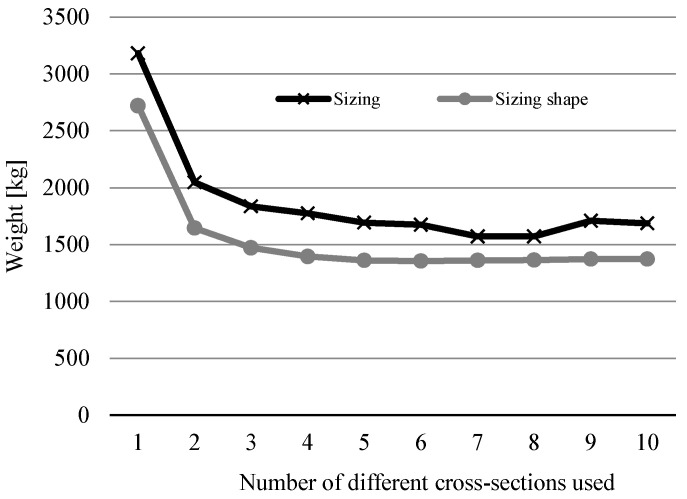
Comparison of results for sizing optimization [14] and sizing shape optimization from this research for different numbers of cross-sections of the 10-bar truss problem.

Figure 5 shows the trend of optimal weight results depending on the number of different cross-sections used for the 10-bar truss example. It compares them to the weights obtained in [14], where only sizing optimization was used with this constraint.

The 17-bar truss example was only optimized for up to 10 different cross-sections. This number was chosen since the optimal solution for this problem uses eight different cross-sections, which is already a very high number, and further increasing the number of different cross-sections would not yield significant results.

Table 4 shows the optimal weights and differences from the solution with a single cross-section and from the optimal solution for the 17-bar truss sizing and shape problem. Table 5 gives the cross-section areas of the optimal models for the 17-bar truss sizing and shape optimization according to the number of different cross-sections used.

The optimal coordinates of points 1 to 9 are given in Table 6 for each solution with a different number of cross-sections. In this example, the x coordinate of point 9 is not a variable, as moving this point in the x direction would decrease the total length of the structure, and this is not considered for this particular example.

As was the case with the 10-bar truss example, coincidentally, the 17-bar truss example also gives the lowest optimal weight using eight different cross-sections.

**Figure 6:**
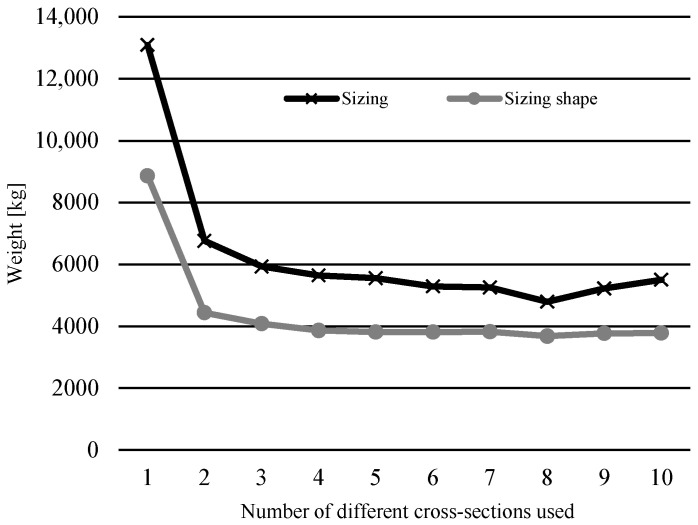
Comparison of results for sizing optimization [14] and sizing shape optimization from this research for different numbers of cross-sections of the 17-bar truss problem.

Figure 6 shows the trend of optimal weight results depending on the number of different cross-sections used for the 17-bar truss example and compares them to the weights achieved in [14]) where only sizing optimization was used with this new constraint. As was expected, the addition of simultaneous shape optimization resulted in a greater decrease in overall weight for all numbers of different cross-sections used.

It is interesting to note that the optimal weights for solutions using more different cross-sections are negligibly greater than the optimal solution. At the same time, the sizing optimization results show a more considerable variance in weight around the optimal number of cross-sections.

For the 25-bar truss sizing and shape problem, Table 7 shows the optimal weights and differences between the solution with a single cross-section and the optimal solution. The key difference with this example, aside from it being a spatial problem, is that the cross-sections are grouped, so multiple bars in certain sections are already grouped, and the number of different cross-sections in any of the eight cross-section groups is being considered.

The optimal coordinates of the points, which are also grouped according to symmetry in this example, are given in Table 8, which gives the cross-section areas of the optimal models for the 25-bar truss sizing and shape optimization according to the number of different cross-section groups used. The number of different cross-sections in groups used for the optimal solution in this case is 6. Table 9 presents the optimal coordinates for each solution with a different number of cross-section groups.

**Figure 7:**
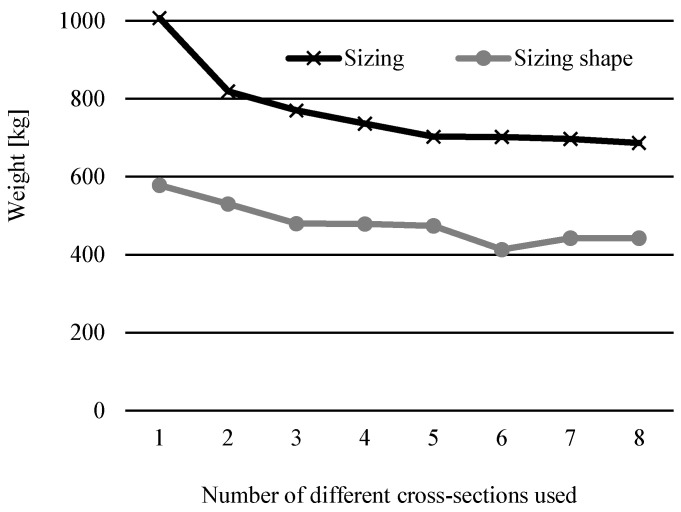
Comparison of results for sizing optimization [14] and sizing shape optimization from this research for different numbers of cross-section groups of the 25-bar truss problem.

Figure 7 shows the trend of optimal weight results depending on the number of different cross-sections used for the 25-bar spatial truss example. It compares them to the weights achieved in [14], where only sizing optimization was used with this new constraint. Once again, the addition of simultaneous shape optimization resulted in a greater decrease in weight overall for all numbers of different cross-sections used.

**Figure 8:**
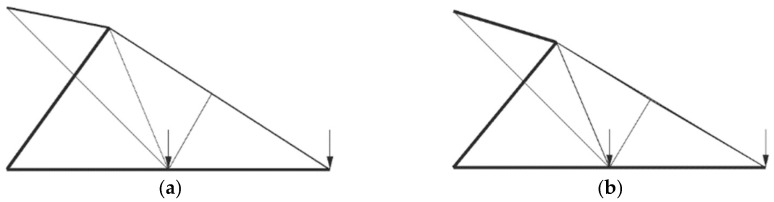
Optimal solutions of the 10-bar truss problem where (**a**) 8 and (**b**) 3 different cross-sections are used.

**Figure 9:**
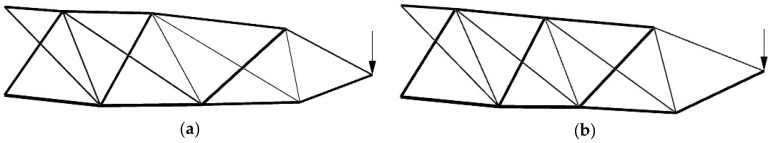
Optimal solutions of the 17-bar truss problem where (**a**) 6 and (**b**) 3 different cross-sections are used.

**Figure 10:**
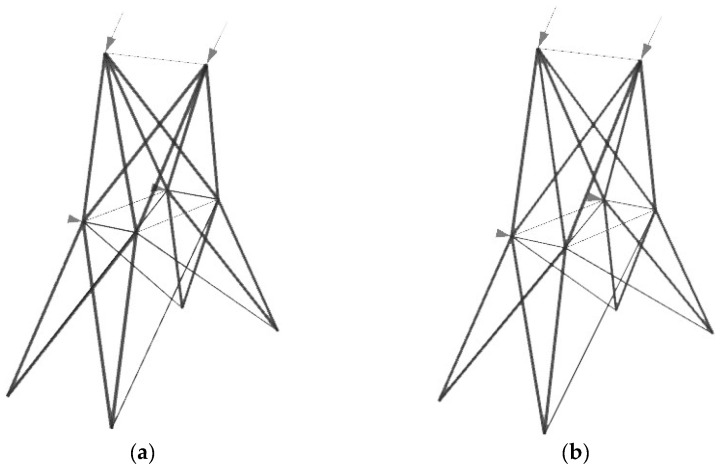
Optimal solutions of the 25-bar spatial truss problem where (**a**) 6 and (**b**) 3 different cross-sections are used in groups.

The optimal structure shapes result from limiting the number of different cross-sections that differ from the initial model. Depending on the number of shape variables, the changes are more drastic between solutions with different numbers of cross-sections with a larger number of variables. In practice, trusses of these and similar shapes would be analytically calculated to have three to four different cross-sections. Figure 8, Figure 9 and Figure 10 show the overall optimal solutions without limiting the number of cross-sections and the solutions with only three different cross-sections.

Visually, there is a small difference in the optimal and three cross-section solutions for the 10-bar truss problem. The optimal shape seems to be with node (1) along bar 9. This suggests that topological optimization could potentially improve upon this solution by eliminating doubled elements along the position of bar 9. The bars in compression/tension are the same in this shape configuration.

All the solutions of the 17-bar truss, except the solution that uses one cross-section for all the bars, have the position of nodes in the bottom row (3, 5, and 7) below 0 and node (7) above its initial position.

**Figure 11:**
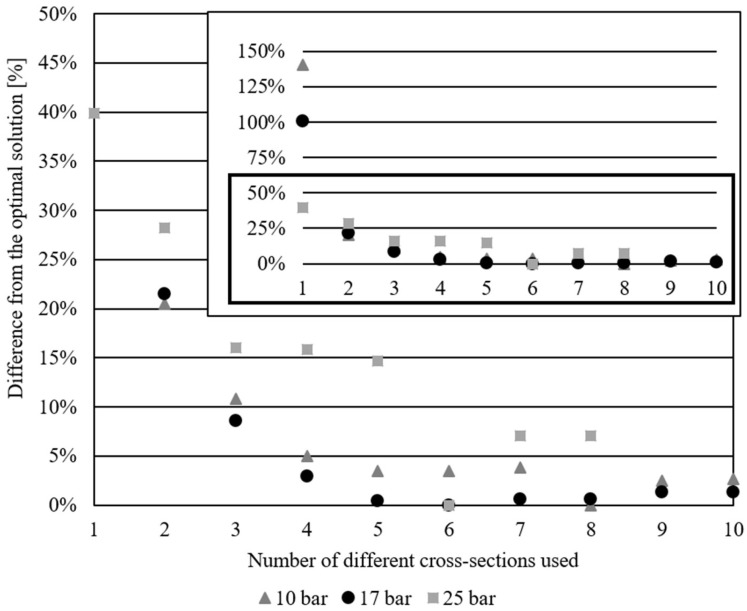
Differences from the optimal solutions based on the number of different cross-sections used for 10, 17 and 25-bar examples.

Figure 11 shows the differences in % from the respective overall optimal solutions, based on the number of different cross-sections used, for all three examples to illustrate the trend of weight decrease. The graph is shown with all values and a scaled version of the below 50% values to better illustrate the variations that are too close in the image when the graph is in the 0–150% range.

## 6. Conclusions

This research results from the efforts made by the authors towards a broader set of goals in trying to find ways to bring the optimization of truss structures closer to the practical problems they represent. In previous years, significant steps were taken to add standard constraints which better represent the actual engineering constraints in designing trusses. The first step towards bringing the truss optimization problem closer to applicable solutions was the implementation of discrete variables. The next major issue was adding a dynamic buckling constraint for compressed bars to avoid structures that would buckle under the load. The addition of each new constraint resulted in drastic increases in the problem’s complexity and the discontinuation of the non-linear, non-convex, and implicit search space.

By constraining the number of different cross-sections used, this research shows that there is a possibility of obtaining better results than through the conventional design methods while using a reasonable number of different cross-sections. The use of a large number of different cross-sections unnecessarily increases the complexity of the designed structure and makes it impractical for on-site assembly, as well as for logistics. The use of a large number of different cross-sections is also wasteful as there are more different cross-sections of unused stock when cutting the bars to size. Using a smaller number of different cross-sections simplifies the structure, as well as decreasing the possibility of human error during the cutting and assembly process.

This paper examines the consequences of using all the aforementioned constraints on typical test examples with 10, 17, and 25 bars. Between one and four different cross-sections are used, in practice, for most truss designs. The results for many different numbers of different cross-sections (except for the 17-bar example where the maximum number was 10 for practical reasons) are presented in this paper for all three examples. As a visual representation of the practical results, the figures are shown for overall optimal solutions (without the maximum number of different cross-sections constraint) and correspondingly, limited to three different cross-sections.

Two main parameters were compared in this research: the difference in weight from the solution, which uses the same cross-section for all the bars, and the difference in weight from the overall optimal solution. The 10-bar truss has an overall optimal weight of 3685.142 kg using ten different cross-sections, the 17-bar 1355.876 kg using six different cross sections, and the 25-bar 413.612 kg using six different cross-sections in 8-bar groups. The planar truss results, which use four different cross-sections, weigh less than 5% more than their corresponding overall optimal solutions, while using three different cross sections results in a ~10% increase (10.847% for the 10-bar, and 8.541% for the 17-bar example). The 25-bar truss is a specific problem, more than just a spatial problem, as the bars in this example are grouped so that each group has the same cross-section. It is hypothesized that the reason this example does not follow the same trend in weight decrease, according to the number of different cross-sections used, is due to this grouping of elements, as well as the limited variability of node positions. The weight of three and four different cross-section models for the 25-bar example do not vary by a lot; therefore, using three instead of four different cross-sections in this example would result in an increase of 0.118% from the four different cross-sections, and 16.037% from the overall optimal solution.

All the examples have less than a 20% increase in weight compared to the optimal for solutions with three and four different cross-sections (less than 11% for planar trusses). This means that using the *m_max_* < 3 or *m_max_* < 4 constraints would not result in drastic increases in weight, but would result in practically applicable results.

Results from this research were also compared to the corresponding results of the just sizing optimization of the same examples, according to the number of different cross-sections used, taken from previous work. The simultaneous addition of shape optimization resulted in an average decrease in weight of optimal results of approximately 30% across all corresponding 10-bar truss solutions with different numbers of different cross-sections used; approximately 18% for the 17-bar truss problem, and approximately 37% average decrease for the 25-bar problem. By using the sizing and shape optimization, the weight of trusses limited to three different cross-sections were ~31% less compared to only sizing optimized for the 10-bar truss, ~20% for the 17-bar truss, and ~38% for the 25-bar truss, while decreases when using four different cross-sections were approximately 31%, 21%, and 35%, respectively.

Once the optimal number of different cross-sections is greater than that of the global optimal number, the weight of the structure gradually increases. This is most likely due to the algorithm being forced to use that particular number, and therefore, one or more bars have to be oversized. The increase is insignificant; however, since these results are practically inapplicable, their purpose is just to show that the global optimal number of different cross-sections is achieved without the cardinality constraint.

The simultaneous optimization of sizing and shape yields results with a lower overall weight than when just using sizing optimization, leading to a more economical solution overall. Weight optimization contributes not only to the direct cost savings of material used but also to other aspects such as transport, logistics, surface coverage, etc. It is evident from the results presented here that there is a need to include topological optimization simultaneously with this process. From observing the results, it can be concluded that there are bars in all examples which have unused or minimally loaded bars, which could be eliminated to save additional weight. This will be the goal of the authors’ future research.

## Figures and Tables

**Table 1:** Cross-section areas of optimal models for 10-bar truss sizing and shape optimization.

Element No.	Cross-Section Areas According to the Number of Different Cross-Sections Used, cm^2^									
	1	2	3	4	5	6	7	8	9	10
1	415.476	380.133	380.133	213.825	176.715	176.715	176.715	176.715	176.715	176.715
2	415.476	56.745	56.745	63.617	44.179	44.179	50.265	33.183	122.718	132.732
3	415.476	380.133	380.133	380.133	380.133	380.133	380.133	380.133	415.476	415.476
4	415.476	380.133	380.133	380.133	380.133	380.133	380.133	346.361	380.133	380.133
5	415.476	56.745	56.745	63.617	44.179	44.179	44.179	28.274	1.131	1.131
6	415.476	56.745	56.745	63.617	44.179	44.179	44.179	28.274	23.758	23.758
7	415.476	56.745	1.131	1.131	1.131	1.131	1.131	1.131	56.745	56.745
8	415.476	380.133	380.133	380.133	380.133	380.133	380.133	380.133	226.980	226.980
9	415.476	56.745	56.745	63.617	95.033	95.033	95.033	95.033	122.718	122.718
10	415.476	56.745	1.131	1.131	1.131	2.011	2.011	2.011	7.069	7.069
Weight, kg	8866.484	4440.629	4084.884	3869.295	3812.595	3813.71	3824.721	3685.142	3775.581	3781.66

**Table 2:** Optimal weights and differences for the 10-bar truss sizing and shape problem.

No. of Different Cross-Sections	Weight, kg	Difference from the Solution with a Single Cross-Section, %	Difference from the Optimal Solution, %
1	8866.484	-	140.601
2	4440.629	49.917	20.501
3	4084.884	53.929	10.847
4	3869.295	56.360	4.997
5	3812.595	57.000	3.459
6	3813.71	56.987	3.489
7	3824.721	56.863	3.788
8	3685.142	58.437	-
9	3775.581	57.417	2.454
10	3781.66	57.349	2.619

**Table 3:** Optimal coordinates of points according to the number of different cross-sections used for the 10-bar truss example.

Number of Cross-Sections Used	Coordinate			
	*x*_1_	*y*_1_	*x*_3_	*y*_3_
1	11.487	1.427	11.312	3.126
2	11.561	3.172	6.817	7.235
3	11.524	3.963	6.037	7.326
4	11.635	3.97	5.957	7.405
5	11.600	3.975	5.916	7.452
6	11.600	3.972	5.916	7.452
7	11.600	3.972	5.916	7.452
8	11.596	4.284	5.789	8.003
9	10.230	3.516	8.888	5.305
10	10.230	3.516	8.888	5.305

**Table 4:** Optimal weights and differences for the 17-bar truss sizing and shape problem.

No. of Different Cross-Sections	Weight, kg	Difference from the Solution with a Single Cross-Section, %	Difference from the Optimal Solution, %
1	2720.745	-	100.663
2	1647.07	39.463	21.476
3	1471.678	45.909	8.541
4	1395.838	48.696	2.947
5	1361.614	49.954	0.423
6	1355.876	50.165	-
7	1363.586	49.882	0.569
8	1364.098	49.863	0.606
9	1373.841	49.505	1.325
10	1373.769	49.508	1.320

**Table 5:** Cross-section areas of optimal models for 17-bar truss sizing and shape optimization.

Element No.	Cross-Section Areas According to the Number of Different Cross-Sections Used, cm^2^									
	1	2	3	4	5	6	7	8	9	10
1	86.590	86.590	56.745	56.745	63.617	56.745	56.745	56.745	56.745	56.745
2	86.590	3.801	15.904	15.904	12.566	19.635	15.904	15.904	15.904	15.904
3	86.590	86.590	86.590	86.590	86.590	86.590	95.033	95.033	95.033	95.033
4	86.590	3.801	15.904	15.904	12.566	19.635	15.904	15.904	15.904	15.904
5	86.590	86.590	56.745	56.745	63.617	56.745	56.745	56.745	56.745	56.745
6	86.590	3.801	15.904	15.904	12.566	19.635	15.904	15.904	15.904	15.904
7	86.590	86.590	86.590	86.590	63.617	78.540	78.540	86.590	86.590	86.590
8	86.590	3.801	15.904	15.904	1.767	4.909	6.158	7.069	8.042	8.042
9	86.590	86.590	56.745	44.179	50.265	44.179	44.179	38.485	38.485	38.485
10	86.590	3.801	15.904	15.904	1.767	4.909	6.158	7.069	8.042	7.069
11	86.590	86.590	56.745	56.745	63.617	56.745	63.617	63.617	63.617	63.617
12	86.590	3.801	15.904	15.904	1.767	4.909	6.158	7.069	8.042	7.069
13	86.590	86.590	15.904	15.904	50.265	19.635	15.904	15.904	15.904	15.904
14	86.590	86.590	56.745	44.179	50.265	44.179	44.179	38.485	33.183	33.183
15	86.590	86.590	56.745	44.179	50.265	44.179	44.179	44.179	44.179	44.179
16	86.590	3.801	56.745	44.179	50.265	44.179	44.179	44.179	44.179	44.179
17	86.590	86.590	56.745	56.745	50.265	56.745	56.745	56.745	56.745	56.745
Weight, kg	2720.745	1647.07	1471.678	1395.838	1361.614	1355.876	1363.586	1364.098	1373.841	1373.769

**Table 6:** Optimal coordinates of points according to the number of different cross-sections used for the 17-bar truss example.

Coordinate	Number of Cross-Sections Used									
	1	2	3	4	5	6	7	8	9	10
*x*_3_	2.652	2.722	2.741	2.65	2.599	2.651	2.664	2.599	2.562	2.563
*y*_3_	0.123	−0.247	−0.269	−0.278	−0.342	−0.303	−0.301	−0.301	−0.288	−0.256
*x*_4_	3.12	1.524	1.532	1.614	1.518	1.604	1.613	1.615	1.677	1.706
*y*_4_	2.125	2.498	2.436	2.41	2.455	2.411	2.418	2.418	2.408	2.405
*x*_5_	5.192	5.676	4.99	5.297	4.922	5.45	5.516	5.525	5.749	5.756
*y*_5_	0.373	−0.136	−0.285	−0.295	−0.236	−0.272	−0.279	−0.275	−0.2	−0.199
*x*_6_	5.184	3.993	4.029	3.983	3.935	4.068	4.071	4.071	3.897	3.917
*y*_6_	1.881	1.833	2.194	2.231	2.356	2.356	2.417	2.421	2.4	2.415
*x*_7_	6.665	6.949	7.699	8.097	7.682	8.16	8.232	8.289	8.392	8.35
*y*_7_	0.296	−0.144	−0.445	−0.327	−0.111	−0.205	−0.228	−0.191	0.025	−0.004
*x*_8_	6.649	6.385	7.085	7.488	7.306	7.766	7.791	7.791	8.197	8.281
*y*_8_	1.917	1.338	1.927	1.921	1.815	1.909	1.929	1.939	1.947	2.015
*y*_9_	0.828	0.343	0.718	0.748	0.327	0.584	0.704	0.769	0.816	0.835

**Table 7:** Optimal weights and differences for the 25-bar truss sizing and shape problem.

No. of Different Cross-Section Groups	Weight, kg	Difference from the Solution with a Single Cross-Section, %	Difference from the Optimal Solution, %
1	578.458	-	39.855
2	530.288	8.327	28.209
3	479.944	17.030	16.037
4	479.164	17.165	15.849
5	474.359	17.996	14.687
6	413.612	28.497	-
7	442.939	23.428	7.090
8	442.895	23.435	7.080

**Table 8:** Cross-section areas of optimal models for 25-bar truss sizing and shape optimization.

Element Group No.	Cross-Section Group Areas According to the Number of Different Cross-Sections Used, cm^2^							
	1	2	3	4	5	6	7	8
1	28.274	12.566	1.131	4.909	4.909	1.131	1.131	2.011
2	28.274	33.183	33.183	28.274	28.274	23.758	23.758	23.758
3	28.274	33.183	33.183	28.274	28.274	33.183	33.183	33.183
4	28.274	12.566	1.131	4.909	1.131	1.131	1.131	1.131
5	28.274	12.566	7.069	4.909	4.909	4.909	4.909	3.142
6	28.274	12.566	7.069	15.904	15.904	7.069	7.069	7.069
7	28.274	33.183	33.183	28.274	28.274	28.274	28.274	28.274
8	28.274	33.183	33.183	33.183	33.183	28.274	38.485	38.485
Weight, kg	578.458	530.288	479.944	479.164	474.359	413.612	442.939	442.895

**Table 9:** Optimal coordinates of points according to the number of different cross-sections used for the 25-bar truss example.

Node Coordinates				
No. of Different Cross-Section Groups	−x_3_, x_4_, x_5_, −x_6_ [m]	y_3_, y_4_, −y_5_, −y_6_ [m]	z_3_, z_4_, z_5_, z_6_ [m]	−x_7_, x_8_, x_9_, −x_10_ [m]
1	1.016	2.374	4.888	2.032
2	1.016	2.232	5.506	2.032
3	1.016	2.388	5.278	2.032
4	1.016	2.29	5.132	2.032
5	1.016	2.26	5.136	2.032
6	1.016	2.6	4.86	2.032
7	1.016	2.734	4.572	2.032
8	1.016	2.684	4.672	2.032

## Data Availability

Data are contained within the article.
